# Prevalence of *Trypanosoma cruzi* infection among Bolivian immigrants in the city of São Paulo, Brazil

**DOI:** 10.1590/0074-02760160384

**Published:** 2016-11-16

**Authors:** Expedito JA Luna, Celia R Furucho, Rubens A Silva, Dalva M Wanderley, Noemia B Carvalho, Camila G Satolo, Ruth M Leite, Cassio Silveira, Lia MB Silva, Fernando M Aith, Nivaldo Carneiro, Maria A Shikanai-Yasuda

**Affiliations:** 1 Universidade de São Paulo, Instituto de Medicina Tropical, São Paulo, SP, Brasil; 2 Hospital das Clínicas da Faculdade de Medicina da Universidade de São Paulo, São Paulo, SP, Brasil; 3 Secretaria Estadual de Saúde de São Paulo, Superintendência de Controle de Endemias, São Paulo, SP, Brasil; 4 Irmandade da Santa Casa de Misericórdia de São Paulo, Centro de Saúde Escola Prof Alexandre Vranjac, São Paulo, SP, Brasil; 5 Secretaria Estadual de Saúde de São Paulo, Centro de Vigilância Epidemiológica, São Paulo, SP, Brasil; 6 Faculdade de Ciências Medicas da Santa Casa de São Paulo, Departamento de Medicina Social, São Paulo, SP, Brasil; 7 Universidade de São Paulo, Faculdade de Medicina, Departamento de Medicina Preventiva, São Paulo, SP, Brasil; 8 Universidade de São Paulo, Faculdade de Medicina, Departamento de Doenças Infecciosas e Parasitárias, São Paulo, SP, Brasil

**Keywords:** Trypanosoma cruzi, Chagas disease, seroepidemiologic studies, emigrants and immigrants, Brazil, Bolivia

## Abstract

With the urbanisation of the population in developing countries and the process of globalisation, Chagas has become an emerging disease in the urban areas of endemic and non-endemic countries. In 2006, it was estimated that the prevalence of Chagas disease among the general Bolivian population was 6.8%. The aim of the present study was to determine the prevalence of *Trypanosoma cruzi* infection among Bolivian immigrants living in São Paulo, Brazil. This study had a sample of 633 volunteers who were randomly selected from the clientele of primary care units located in the central districts of São Paulo, Brazil. Infection was detected by two different ELISA assays with epimastigote antigens, followed by an immunoblot with trypomastigote antigens as a confirmatory test. The prevalence of the infection was 4.4%. Risk factors independently associated with the infection were: a history of rural jobs in Bolivia, knowledge of the vector involved in transmission, and having relatives with Chagas disease. Brazil has successfully eliminated household vector transmission of *T. cruzi*, as well as its transmission by blood transfusion. The arrival of infected immigrants represents an additional challenge to primary care clinics to manage chronic Chagas disease, its vertical transmission, and the blood derivatives and organ transplant programs.

The World Health Organization estimates that between 7 and 8 million people are infected by *Trypanosoma cruzi*, the etiologic agent of Chagas disease (WHO 2013). Rural areas of 21 countries of the Latin American sub-continent from Mexico to the Southern Cone, are the traditionally recognised endemic regions for Chagas disease. Progress in vector transmission control, as well as in blood borne transmission control in endemic countries, have gradually changed the epidemiology of the disease. The Pan American Health Organization has certified the elimination of household transmission of the parasite from Uruguay (1997), Chile (1999), Brazil (2006), and Paraguay (2013); however, household transmission still occurs in the other countries of the Latin American sub-continent (PAHO 2014). Brazil has eradicated its main domiciliary vector, the *Triatoma infestans*. Extensive serosurveys among children have confirmed the elimination of transmission. In the state of São Paulo, elimination of transmission was achieved in the 1970s (Carvalho et al. 2011, Ostermayer et al. 2011).

In addition to the successful elimination of household vector transmission, Brazil has greatly reduced, if not eliminated, transmission by blood and blood product transfusion, and by organ and tissue donation. Mandatory screening of blood and organ donors has become mandatory since 1994. However, the zoonotic transmission cycle still occurs, involving wild rodents, marsupials and other mammals of the South American fauna. Humans may accidentally be exposed to the zoonotic cycle while conducting outdoors activities in forest areas, such as camping, fishing, or logging, or by the consumption of contaminated food, particularly wild fruit juices (OPAS 2009). Between 2004-2014, over 1700 cases of acute Chagas disease were reported in 123 outbreaks, mainly in the Brazilian Amazon. Approximately 100-150 new cases of acute Chagas disease are reported every year in Brazil. The vast majority of these cases are related to foodborne transmission through the consumption of products containing non-pasteurised “açaí” or “bacaba”, Amazonian regional fruits, contaminated with infected triatominae or with secretions from the anal glands of marsupials (OPAS 2009, MS/SVS 2015).

Migration of chronically infected individuals has brought the disease to some developed countries in North America, Western Europe and Asia (Schmunis & Yadon 2010). Within South America, migrants continuously leave rural areas for the cities, a movement that may potentially include Chagas carriers. The city of São Paulo is the largest city in South America. It attracts migrants from all over the Latin American sub-continent. It is estimated that 300,000 Bolivian immigrants live in the city. The prevalence of the infection in Bolivia has been estimated at 6.8% (OPS 2006). This large population from a region where the infection remains endemic may pose an additional burden on the Brazilian public health system and on national blood and organ donation programs. The aim of the present study was to assess the prevalence of *T. cruzi* infection in a sample of Bolivian immigrants living in downtown São Paulo and attending primary care clinics located in central São Paulo. This study is part of a larger research project designed to understand the access to health care by the Bolivian immigrant community and to produce guidelines for Chagas disease management in primary care settings.

## MATERIALS AND METHODS

A seroprevalence survey of *T. cruzi* infection among the immigrant Bolivian clientele of the primary care clinics of the Brazilian Public Health System (SUS), located in districts of downtown São Paulo, was undertaken.

A sample of 633 volunteers was randomly selected from the clinics patient registry, taking into account an expected prevalence rate of 0.07 with an acceptable error of 0.014 and adding 10% to the sample size to compensate for potential participant losses. The sample included 111 children < 10 years of age.

*Serological methods* - Infection with *T. cruzi* was defined as a positive result by two serological tests with parasite antigens.

Two commercialised ELISA - based serological tests (Chagas test ELISA III, Bioschile Ingenieria Genetica SA, Santiago, Chile) and ELISA cruzi (BioMerieux Diagnostics SA, Rio de Janeiro, Brazil), which detect antibodies against *T. cruzi* antigens attached to the inner surfaces of microplate wells, were employed according to manufacturer instructions. These tests were repeated in case of discrepant results (positive and negative, or doubtful test result). Confirmatory tests were performed by Tesablot (Umezawa et al. 1996) and recombinant ELISA with trypo and epimastigote antigens (ELISA Chagatest® Wiener Lab, Rosario, Argentina), according to manufacturer instructions.

Sera from the test sample, and positive and negative controls, were applied and incubated at room temperature for 30 min. After washing, chromogenic substrate goat was added and then the reaction was stopped, by adding 1 *N* sulphuric acid. After the addition of the enzyme substrate, the reaction was stopped with stop solution. A positive reaction was defined according to the manufacturer instructions. The reading was performed using a 450 nm filter and a 620-630 nm reference filter.

For immunoblot with trypomastigote antigens, antigen prepared from supernatants of cell culture containing trypomastigotes (Umezawa et al. 1996) were fractionated by polyacrylamide gel electrophoresis (Mini-Protean II; BioRad, Berkeley, CA) and electrophoretically transferred to a nitrocellulose membrane with a porosity of 0.45 µM (BioRad, Berkeley, CA) in a semi-dry system (Hoefer Scientific Instruments, San Francisco, CA). Sera were diluted and incubated for 2 h at room temperature. Antibodies were detected by addition of anti-human IgG labelled with peroxidase (Sigma Chemical St. Louis, MO) and incubated for 1 h and 30 min at room temperature. After the development of the immune-complex by adding 600 μL of solution containing H_2_O_2_, 4-cloro1-naphthol (Sigma Chemical St. Louis, MO), diluted in methanol, the reaction was stopped by adding deionised water. The sample was considered positive when the band of approximately 160kDa was revealed.

*Ethics statement* - The study was conducted in accordance to the principles of the Helsinki Declaration and Brazilian ethical regulations. Selected individuals received a home visit to explain the project and invite them to participate. Those who agreed to participate were asked to sign an informed consent form, answer a questionnaire, and provide a blood sample for analysis. Parents or legal guardians of participants < 18 years of age provided the informed consent on their behalf. Questionnaires and consent forms were translated into Spanish, and interviews were conducted in Spanish, by Spanish-speaking community health agents. Participants with a diagnosis of *T. cruzi* infection were referred to the primary care clinics of the Brazilian Public Health System for further diagnostic investigation and treatment.

The study protocol was submitted and approved by the research ethics committee of the “Hospital das Clínicas” of the University of São Paulo Medical School (CAPPESQ no. 196.698/2013).

## RESULTS

The characteristics of the population sample are presented in Table I. The majority of the volunteers were young adults, living in São Paulo for < 5 years, and were born in the Department of La Paz, Bolivia. Of these, almost 50% reported having lived in the rural areas of Bolivia, and 33.3% had undertaken rural jobs there. A very small proportion of participants were aware of the insect vector causing Chagas disease and reported having been bitten by it.

**TABLE I:** Characteristics of the study sample, São Paulo (SP), Brazil, 2014

Variable	(nº)	(%)
Gender		
Male	293	46.3
Age		
< 10	105	16.6
10 - 19	85	13.4
20 - 29	222	35.1
30 - 39	154	24.3
40 and more	67	10.6
Time living in SP		
0 - 1 year	136	21.5
2 - 5 years	269	42.5
6 - 10 years	102	16.1
11 and more	126	19.9
Country of birth		
Brazil	87	13.7
Other	7	1.1
Bolivia	539	85.2
Dept. La Paz	410	64.8
Exposure to Chagas disease and knowledge about it		
Lived in rural zone in Bolivia	309	48.8
Worked in rural jobs in Bolivia	211	33.3
Knows the “vichunca”	30	4.7
Found it in household	232	36.7
Has been bitten by “vichunca”	30	4.7
Has relatives with Chagas	51	8.1

The prevalence of *T. cruzi* infection was 4.4% (95% confidence interval: 2.8 - 6.0). The distribution of the population sample, according to the variables examined in this study and their association with Chagas infection, is presented in Table II.

**TABLE II:** Distribution of Chagas infection, according to variables examined in this study, São Paulo (SP), Brazil, 2014

Variables/Categories	*Trypanosoma cruzi* infection - n (%)		p

	Negative	Positive	
Sex			0.13 *
Male	284 (96.9)	9 (3.1)	
Female	321 (94.4)	19 (5.6)	
Age group			0.18 **
1 - 9	102 (97.1)	3 (2.9)	
10 - 19	83 (97.6)	2 (2.4)	
20 - 29	210 (94.6)	12 (5.4)	
30 - 39	148 (96.1)	6 (3.9)	
40 and more	62 (92.5)	5 (7.5)	
Time living in SP			0.49 **
0 - 1 year	127 (93.4)	9 (6.6)	
2 - 5	261 (97.0)	8 (3.0)	
6 - 10	95 (93.1)	7 (6.9)	
11 and more	122 (96.8)	4 (3.2)	
Country of birth			0.02***
Bolivia	511 (94.8)	28 (5.2)	
Brazil and others	94 (100.0)	-	
Department of birth (for those born in Bolivia)			< 0.01*
La Paz	403 (98.3)	7 (1.7)	
Other	108 (83.7)	21 (16.3)	
Lived in rural areas in Bolivia			0.12*
Yes	291 (94.2)	18 (5.8)	
No	252 (96.9)	8 (3.1)	
Worked in rural jobs in Bolivia			0.05*
Yes	197 (93.4)	14 (6.6)	
No	340 (96.9)	11 (3.1)	
Characteristics of house in Bolivia			0.38*
Cement	90 (98.9)	1 (1.1)	
Clay	258 (95.6)	12 (4.4)	
Wood	5 (100.0)	-	
Bricks with plaster	219 (94.0)	14 (6.0)	
Bricks without plaster	33 (97.1)	1 (2.9)	
Knows Chagas disease			< 0.01*
Yes	160 (90.4)	17 (9.6)	
No	445 (97.6)	11 (2.4)	
Knows the “vichunca”			< 0.01*
Yes	210 (90.5)	22 (9.5)	
No	395 (98.5)	6 (1.5)	
Has been bitten by the “vichunca”			0.05***
Yes	26 (86.7)	4 (13.3)	
No	416 (96.1)	17 (3.9)	
Has found the “vichunca” in/around the household			< 0.01*
Yes	102 (90.3)	11 (9.7)	
No	373 (97.1)	11 (2.9)	
Has relatives with Chagas			< 0.01***
Yes	49 (79.0)	13 (21.0)	
No	483 (97.6)	12 (2.4)	
Has received blood transfusion			0.51*
Yes	27 (93.1)	2 (6.9)	
No	578 (95.7)	26 (4.3)	

*: pearson chi-square; **: chi-square for linear trend; ***: Fisher exact test.

In univariate analysis, variables related to the place of birth were significantly associated with infection. Participants born in Bolivia had an increased risk of infection, compared to participants born in Brazil or another country. Of note, participants born in Brazil or in other countries were mainly children. For participants born in Bolivia, birth in the Department of La Paz was a protective factor, compared to all other Bolivian departments. No participants born in Brazil or in other countries presented the serologic markers of infection. A history of rural jobs in Bolivia was also significantly associated with infection.

Prevalence of infection among women was higher than among men, although the difference was not significant. Except for two cases, all infected women were of childbearing age.

Participants with some knowledge of Chagas disease and those with knowledge of the insect vector had an increased risk of infection. The participants who reported finding the insect vector in or around their home in Bolivia or having been bitten by it were also at increased risk of infection. Furthermore, having relatives with Chagas disease was significantly associated with an increased risk of infection.

Variables associated with infection, with a p value < 0.10, were analysed using a multivariate logistic regression model in order to identify the variables independently associated with an increased risk of infection (Table III). The independent risk factors for infection were: having undertaken rural jobs in Bolivia, knowledge of the vector, and having relatives with Chagas disease. When comparing the birth place of participants, according to the departments in Bolivia, being born in the Department of La Paz was a protective factor.

**TABLE III:** Logistic regression analysis of factors independently associated with *Trypanosoma cruzi* infection, São Paulo (SP), Brazil, 2014

Variable	p	Exp β	95% CI Exp β	
Born in La Paz Department	< 0.001	0.127	0.044	0.369
Worked in rural jobs in Bolivia	0.023	3.163	1.176	8.504
Knows the “vichunca”	0.024	3.749	1.189	11.822
Has relatives with Chagas	0.018	3.412	1.239	9.398

CI: confidence intervals

## DISCUSSION

This is the first serosurvey of *T. cruzi* infection conducted among the Bolivian immigrant community in Sao Paulo, Brazil. The prevalence of infection was slightly lower than the previously reported prevalence among the Bolivian population. Individuals who migrate tend to be younger and healthier than those who do not. Infection was not associated with sex or age. It is noteworthy that three children < 10 years of age were infected. Two of these were twin brothers; their mother was also infected. Taking into consideration the period that they lived in Brazil, vector transmission in Bolivia, before they moved, could not be excluded.

Knowledge of the “vichunca”, the popular name for the insect vector in Bolivia, having relatives with Chagas disease, and a history of rural jobs in Bolivia, were risk factors independently associated with infection. It is likely that these variables indicate proximity to the transmission sites of the parasite. Most of the participants were from the Department of La Paz, where Chagas prevalence is lower than in the other departments of the country. Being born in La Paz Department was a protective factor for infection. Although there are no statistical data on the specific department of origin of Bolivian immigrants, social scientists have highlighted that a large proportion of the immigrant community living in São Paulo came from the Department of La Paz and, specifically, from El Alto, Bolivia’s second largest city, located on the outskirts of the capital city. At the start of migration to São Paulo, a sewing industry in El Alto provided experience which later helped the immigrants to find jobs in the garment industry (Xavier 2012).

A limitation to our study is that the population sample was selected from Bolivian immigrants living in downtown São Paulo and attending primary care services located in this area. The immigrants who registered with the public health system may have already adapted to their new country and may not, therefore, be representative of the general Bolivian community in the city.

Considering the observed prevalence of infection in this study and the estimated number of Bolivian immigrants in São Paulo, between 8,400 and 18,000 Bolivians could be infected by *T. cruzi*. The prevalence of infection among women of childbearing age was 6.1% and maternal-foetal transmission may occur in 4.9% to 6.0% of pregnant women (Oliveira et al. 2010). This would result in 476 - 583 cases of congenital Chagas disease among the Bolivian immigrant community in São Paulo over the next three decades.

At the same time, it is estimated that approximately 1.2 million Brazilians are chronically infected with Chagas disease. Nationwide, the prevalence of the infection among blood donor candidates is declining, and has remained < 0.3% over the past decade (Ahkavan 2000, Dias et al. 2002, WHO 2015). It could be argued that the arrival of a few thousand chronically infected immigrants does not substantially change the endemic situation in Brazil. However, since the elimination of household vectorial transmission, the chronically infected Brazilian patients have been ageing. Therefore, the number of pregnant women who are carriers of *T. cruzi* tends to decline and so does the number of infected potential blood and organ donors. Thus, the arrival of young adults and children with chronic infection may pose an additional burden on the Brazilian public health system and its supply of blood and organs.
